# Physical Activity at Growth Induces Bone Mass Benefits Into Adulthood – A Fifteen‐Year Prospective Controlled Study

**DOI:** 10.1002/jbm4.10566

**Published:** 2021-11-26

**Authors:** Björn E Rosengren, Jakob Rempe, Lars Jehpsson, Magnus Dencker, Magnus K Karlsson

**Affiliations:** ^1^ Clinical and Molecular Osteoporosis Research Unit, Department of Orthopedics and Clinical Sciences, Skane University Hospital Malmo Lund University Malmo Sweden; ^2^ Department of Orthopedics, Helsingborg Hospital Lund University Helsingborg Sweden; ^3^ Department of Physiology and Clinical Sciences, Skane University Hospital Malmo Lund University Malmo Sweden

**Keywords:** DXA, CLINICAL TRIAL, EXERCISE, FRACTURE PREVENTION, GENERAL POPULATION STUDIES

## Abstract

Daily school physical activity (PA) improves musculoskeletal traits. Whether or not benefits remain in adulthood is debated. We included in this study 131 children that took part in an intervention with 40 minutes of PA per school day (200 minutes per week) from age 6 to 9 years (grade one) to age 14 to 16 years (grade nine), whereas 78 children continued with national recommended school physical education of 60 minutes per week. Measurements were done with dual‐energy X‐ray absorptiometry (bone mineral content [BMC], bone mineral density [BMD], and bone area), and a computerized knee dynamometer (peak torque muscle strength) at study start, at the end of the intervention, and 7 years after the intervention. Group differences from study start and end of the intervention to 7 years thereafter were estimated by analyses of covariance (adjusted for sex and follow‐up time). Musculoskeletal gains from study start to 7 years after termination of the intervention were higher in the intervention group (total body less head BMC +182.5 g [95% confidence interval {CI}, 55.1–309.9] and BMD +0.03 g/cm^2^ [95% CI, 0.003–0.05], femoral neck area + 0.2 cm^2^ [95% CI, 0.1–0.4], and knee flexion peak torque muscle strength at 60 degrees per second +9.2 Nm [95% CI, 2.9–15.5]). There was no attenuation during the 7 years that followed termination of the intervention (all group comparisons *p* > 0.05). Benefits in musculoskeletal gains remain 7 years after termination of a daily school‐based PA program, without attenuation after termination of the program. Daily school PA may counteract low bone mass and inferior muscle strength in adulthood. © 2021 The Authors. *JBMR Plus* published by Wiley Periodicals LLC on behalf of American Society for Bone and Mineral Research.

## Graphical abstract

Gain from mean age 8 to age 23 in total body less head and femoral neck BMC/BMD, femoral neck area and knee flexion peak torque muscle strength (absolute and relative to body weight) in individuals with daily school physical activity expressed in standard deviations (SD), compared to mean gain in the control group (0.0 SD) with the bars representing the 95 percent confidence intervals.

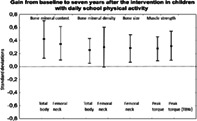

## Introduction

Thirty percent of children suffer fractures,^(^ ^1^ ^)^ and after age 50 years, the same applies to 50% of women and 22% of men,^(^ ^2^ ^)^ resulting in significant suffering and health care burden.^(^ ^3^ ^)^ However, risk factors for fracture have been identified that are addressable by intervention, such as low physical activity (PA).^(^ ^4^, ^5^, ^6^, ^7^, ^8^ ^)^ Because PA is accessible, easy to initiate, without adverse effects, and possible to implement on population‐based level and at all ages, this may constitute an effective strategy for reducing lifelong fracture risk. Studies have also shown that increased PA is associated with benefits in bone mass, bone structure/size, and muscle strength, all traits associated with fracture risk.^(^ ^6^, ^7^, ^8^, ^9^, ^10^, ^11^ ^)^ As 25% of the adult bone mass is acquired during 2 pubertal years^(^ ^12^ ^)^ and the greatest skeletal response to mechanical load occurs during prepubertal and early pubertal years,^(^ ^13^ ^)^ PA interventions early in life probably prove most effective at increasing bone mass in adulthood. Studies have also verified that population‐based PA interventions in these years, with an intensity that allows all children to participate, are associated with musculoskeletal benefits,^(^ ^6^, ^7^, ^8^ ^)^ and low fracture incidence.^(^ ^7^ ^)^

PA school intervention studies in prepubertal and peripubertal children has uniformly shown increased total amount of PA, associated with benefits in bone mass and muscle strength.^(^ ^6^, ^7^, ^8^, ^14^, ^15^ ^)^ A high level of PA in childhood is also associated with high peak bone mass^(^ ^6^, ^7^, ^8^ ^)^ and low fracture incidence in adulthood.^(^ ^16^, ^17^, ^18^, ^19^, ^20^, ^21^ ^)^ This is reasonable, as 50% of the variance in bone mass in old age is predicted by peak bone mass^(^ ^22^ ^)^ and a 10% increase in peak bone mass delays osteoporosis by 13 years.^(^ ^23^ ^)^ Others question this view, reporting that PA‐induced high bone mass is attenuated if the level of activity is reduced.^(^ ^19^, ^24^, ^25^ ^)^ Our recent publication indicated that a school‐based PA program is associated with beneficial effects that may remain into adolescence.^(^ ^26^ ^)^ However, because this study followed the participants for only a short period after the intervention terminated as well as being conflicted by a large drop out frequency, there is a need to repeat the follow‐up with longer retirement period and higher attendance rate. Due to the conflicting results, there is a need for longitudinal, controlled intervention studies that follow individuals before initiation of a PA intervention, through the intervention, and thereafter. These studies should also focus on bone structure/size, because these traits contribute to bone strength and fracture risk independently of bone mass,^(^ ^27^, ^28^ ^)^ and some studies indicate that exercise‐induced benefits in bone mass are lost after reduction in PA, whereas benefits in bone size may be maintained.^(^ ^28^, ^29^, ^30^ ^)^ Muscle strength should also be followed, because it is closely related to PA and an independent predictor of fracture.^(^ ^9^, ^31^ ^)^

We hypothesized that musculoskeletal benefits related to PA in childhood remain in adulthood. As primary research question, we asked whether the musculoskeletal benefits that we found during the intervention with daily school PA^(^ ^6^, ^7^, ^8^ ^)^ remain 7 years after termination of the program, and our secondary question was whether termination of the intervention is associated with attenuation.

## Subjects and Methods

Participants in the current study were collected from the Pediatric Osteoporosis Prevention (POP) study, a study for which previous publications have shown that the children with daily school PA gain a higher bone mass and muscle strength than children with regular school PA.^(^ ^6^, ^7^, ^8^ ^)^ The POP study is a population‐based, prospective, controlled PA intervention study, with the primary aim of investigating whether daily school‐based PA improves musculoskeletal traits. The study includes children from four government‐funded, community‐based, public elementary schools, with children admitted to each school based on domicile. All schools are situated in the same geographical area, with uniform socioeconomic status.

Before study start, all four schools had the same amount of PA (60 minutes per school week). The first school to join became the intervention school, where the amount of PA was increased to 200 minutes per school week (daily school classes of 40 minutes) throughout all nine compulsory school years. PA in Sweden is a compulsory subject, so all children had to participate. The intervention involved an extended time for the regular school PA curriculum, including gymnastics, team sports, running, and jumping. There were no specific extra high‐impact activities included, known to be osteogenic We had no registration as regard the proportion of different activities that were included in the curricula or proportion of impact and endurance exercise. Furthermore, we had no registration to what extent the children participated in the PA lessons, but PA classes are mandatory in Sweden, so all children had to participate. The three control schools continued with the same activities according to the national standard of 60 minutes per school week, provided in one to two classes per school week.

We invited all children that started first grade during 1998–2000 in the four schools to participate. The children were then 6 to 9 years old and 98% were of Caucasian ethnicity. A total of 217 of 237 children (123 boys and 94 girls) in the intervention and 132 of 327 (68 boys and 64 girls) in the control schools accepted the invitation. We excluded four boys and two girls in the intervention and one girl in the control schools with chronic diseases or medication that interfered with bone growth.

**Fig 1:**
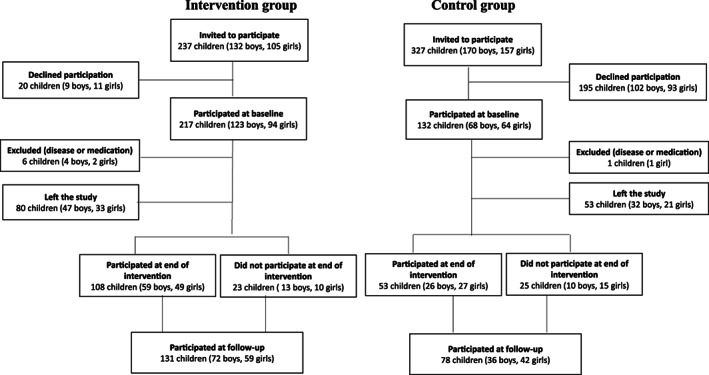
Flowchart of study participants.

In the current study we re‐evaluated the participants 7 ± 2 years (mean ± standard deviation [SD]) after termination of the intervention. Of the 342 children followed from baseline, 57 had moved out of the region, two had died, and one had a serious illness, rendering 282 potential participants. A total of 209 individuals (61% of the children who participated at baseline and 74% of those still living in the region) attended (Fig. 1). The follow‐up evaluation included the same measurements as at baseline and the end of the intervention.^(^ ^6^, ^7^, ^8^ ^)^ A nonvalidated questionnaire evaluated lifestyle, including dairy intake, alcohol, smoking, at least one of the following medical conditions—asthma, achondroplasia, epilepsy, kidney disease, thyroid disease, diabetes, bowel disease—at least one of the following medications—cortisone, levaxin, liothyronine, insulin, antiepileptic drugs, antidepressants, contraceptives—and duration of weekly organized leisure‐time PA. Bone mineral content (BMC; g) and bone mineral density (BMD; g/cm^2^) were measured in total body less head, arms, legs, spine, and left femoral neck, and lean and fat mass (kg) in total body by dual‐energy X‐ray absorptiometry (DXA). We used DPX‐L® version 1.3z (Lunar Corporation, Madison, WI, USA) at baseline and at end of intervention, DXA‐iDXA® version enCore 13.60 (Lunar Corporation) in 188 individuals, and DXA‐Prodigy® version enCore 9.30 (Lunar Corporation) in 21 individuals at the 7‐year postintervention follow‐up. The DXA machines were calibrated daily by a phantom during the entire study period. There was no long‐term drift. Muscle strength was measured as concentric isokinetic peak torque (PT) (Nm) and PT related to total body weight (PTTBW) ([PT per total body weight] × 100) for right knee flexion (_flex_) and right knee extension (_ext_) at a speed of 60 and 180 degrees per second by a computerized dynamometer (Biodex System III Pro®; Biodex Medical Systems Inc., Shirley, NY, USA). We used the highest PT value of five repeated flexion/extension movements. Research technicians performed all measurements. The coefficient of variation (%), evaluated by duplicate measurements in 13 healthy children, was 1.4% to 5.2% for BMC, 2.4% to 2.6% for BMD, 6.7% to 9.1% for PT_flex_, and 6.6% to 12.3% for PT_ext_.

Body height (cm) was measured with a Holtain Stadiometer (Holtain LTD, Pembrokeshire, UK) and body mass (kg) with an electric scale (Avery Berkel HL 120 Electric Scale; Avery Berkel, West Midlands, UK). Body mass index (BMI) was calculated as body weight divided by body height squared (kg/m^2^). A research nurse assessed Tanner stage^(^ ^32^ ^)^ at baseline, whereas self‐assessment was used at follow‐up.

In a first dropout analyses we used the compulsory Swedish first grade school health examinations and compared height, weight, and BMI in the children who agreed to participate at baseline with those who did not. Analyses revealed similar anthropometry; ie, no clinically relevant (or statistically significant) differences between groups.^(^ ^8^ ^)^ In the second dropout analysis we compared age, anthropometry, bone mass, and muscle strength in participants who attended the baseline and the 7‐year postintervention examination with participants who attended baseline but not the 7‐year postintervention examination. In these analyses too, we found no clinically relevant (or statistically significant) differences between groups (Supplemental Appendix 1).

We used Statistica version 12.0 (Statsoft, Inc., Tulsa, OK, USA) for statistical analyses. Descriptive data are presented as absolute numbers (*n*), proportions (%), means with SDs, and inferential statistics as age‐adjusted mean differences with 95% confidence intervals (CIs). Study period changes were estimated during two separate periods: (i) the entire 15‐year period (the 7‐year postintervention values minus baseline values) and (ii) the 7‐year period after termination of the intervention (7‐year postintervention values minus the values at the end of the intervention). Group differences during each of the two periods were evaluated by analysis of covariance (ANCOVA), adjusted for differences in the proportion of boys and girls and the duration of the follow‐up period. Interaction terms (sex and group) were included in order to evaluate whether the intervention effects were different in boys and girls. To express differences between the two groups in SDs (*Z*‐scores) we used the mean ± SD in the control cohort as reference. Sample sizes were at study start (1999) calculated as to be able to find a difference of 0.3 SDs between the intervention and control groups. We regarded *p* < 0.05 as a statistically significant difference. Written consent was obtained before study start from participants and parents/guardians of each participant. The study was approved by the Ethics Committee of Lund University (LU 453‐98; 1998‐09‐15) and registered as a clinical trial (ClinicalTrials.gov NCT000633828).

## Results

### Sex‐specific group characteristics at baseline

Baseline sex‐specific group characteristics are presented in Table 1. All participants were in Tanner stage I at baseline and in Tanner stage V at follow‐up.

**Table 1:** Anthropometry, Lifestyle Characteristics, Soft Tissue Composition, and Musculoskeletal Traits Measured With DXA and Biodex at School Start (Baseline)

Parameter	Boys (*n* = 108)			Girls (*n* = 101)		
	Intervention (*n* = 72)	Control (*n* = 36)	Mean difference (adjusted analysis)	Intervention (*n* = 59)	Control (*n* = 42	Mean difference (adjusted analysis)
Age (years)	7.6 ± 0.6	7.9 ± 0.7	n.a.	7.5 ± 0.6	8.0 ± 0.6	n.a.
Anthropometry						
Height (cm)	128.1 ± 6.5	130.0 ± 6.8	−0.1 (−2.5, 2.3)	126.8 ± 6.2	126.8 ± 6.2	0.7 (−1.7, 3.2)
Weight (kg)	27.5 ± 5.4	27.7 ± 5.6	1.0 (−1.1, 3.2)	26.5 ± 4.9	26.5 ± 4.9	0.2 (−2.1, 2.4)
BMI (kg/m^2^)	16.7 ± 2.4	16.2 ± 1.9	0.7 (−0.3, 1.6)	16.4 ± 2.4	16.4 ± 2.4	0.0 (−0.9, 1.0)
Lifestyle, *n* (%)						
Exclusion of dairy products	1/72 (1%)	3/36 (8%)	n.a.	1/56 (2%)	2/41 (5%)	n.a.
Any medical conditions	9/71 (13%)	2/36 (6%)	n.a.	3/55 (5%)	1/39 (3%)	n.a.
Current medication	3/71 (4%)	0/36 (0%)	n.a.	1/55 (2%)	0/39 (0%)	n.a.
Physical activity (hours/week)						
Total PA	6.2 ± 3.1	4.1 ± 3.0	2.1 (0.8, 3.4)	5.2 ± 1.8	3.2 ± 2.2	2.0 (1.2, 2.9)
Soft tissue composition (kg)						
Total body fat mass	3.8 ± 3.2	3.6 ± 2.5	0.4 (−0.8, 1.7)	4.6 ± 2.6	5.1 ± 3.3	−0.5 (−1.8, 0.8)
Total body lean mass	21.4 ± 2.9	21.9 ± 3.3	0.3 (−0.8, 1.5)	19.4 ± 2.4	20.3 ± 2.6	0.0 (−1.1, 1.0)
BMC (g)						
Total body less head	643.7 ± 147.1	666.1 ± 151.3	19.4 (−35.4, 74.3)	600.8 ± 131.8	629.9 ± 148.9	28.1 (−28.7, 85.0)
Arms	87.9 ± 19.2	88.9 ± 20.5	3.8 (−3.7, 11.48	78.7 ± 17.8	82.2 ± 18.9	2.8 (−4.9, 10.5)
Legs	275.7 ± 68.1	288.6 ± 76.3	8.3 (−17.3, 34.0)	264.3 ± 60.3	283.3 ± 71.4	11.4 (−14.2, 37.1)
Spine	84.3 ± 20.6	85.9 ± 16.9	3.0 (−4.5, 10.5)	80.4 ± 18.4	78.3 ± 16.6	8.2 (0.7, 15.7)
Hip – total hip	15.9 ± 3.3	16.2 ± 5.1	0.1 (−2.1, 2.3)	13.4 ± 2.8	15.3 ± 5.0	−0.6 (−3.3, 2.0)
Hip – femoral neck	2.8 ± 0.6	3.0 ± 0.9	0.0 (−0.3, 0.3)	2.5 ± 0.6	2.7 ± 0.7	0.0 (−0.3, 0.3)
Hip – Wards triangle	1.2 ± 0.3	1.4 ± 0.9	−0.1 (−0.3, 0.2)	1.1 ± 0.5	1.2 ± 0.6	−0.0 (−0.3, 0.2)
BMD (g/cm^2^)						
Total body less head	0.69 ± 0.05	0.70 ± 0.06	0.01 (−0.01, 0.03)	0.68 ± 0.05	0.69 ± 0.05	0.01 (−0.01, 0.03)
Arms	0.62 ± 0.04	0.61 ± 0.05	0.02 (0.00, 0.03)	0.60 ± 0.04	0.60 ± 0.05	0.01 (−0.01, 0.03)
Legs	0.75 ± 0.07	0.76 ± 0.08	0.01 (−0.01, 0.04)	0.74 ± 0.06	0.76 ± 0.07	0.00 (−0.03, 0.03)
Spine	0.68 ± 0.06	0.69 ± 0.05	0.00 (−0.03, 0.02)	0.68 ± 0.06	0.69 ± 0.07	0.00 (−0.03, 0.03)
Hip – femoral neck	0.77 ± 0.11	0.79 ± 0.12	0.01 (−0.04, 0.05)	0.71 ± 0.10	0.71 ± 0.10	0.02 (−0.02, 0.07)
Hip – Wards triangle	0.80 ± 0.13	0.84 ± 0.14	−0.02 (−0.07, 0.04)	0.75 ± 0.14	0.74 ± 0.13	0.02 (−0.04, 0.08)
Bone size (cm^2^)						
Hip – femoral neck	3.6 ± 0.4	3.7 ± 0.8	−0.0 (−0.3, 0.2)	3.5 ± 0.4	3.7 ± 0.5	−0.1 (−0.3, 0.1)
Peak torque muscle strength (Nm)						
Knee extension (60 degrees)	41.1 ± 9.3	43.8 ± 11.6	−0.4 (−3.9, 3.1)	40.7 ± 10.9	45.0 ± 11.0	−0.6 (−5.1, 4.0)
Knee extension (180 degrees)	33.9 ± 7.4	36.2 ± 8.9	−0.1 (−2.9, 2.7)	32.2 ± 8.3	35.9 ± 7.7	−0.9 (−4.3, 2.4)
Knee flexion (60 degrees)	21.9 ± 6.9	24.9 ± 7.1	−1.4 (−4.0, 1.3)	19.8 ± 5.3	24.3 ± 5.6	−3.6 (−5.5, −0.8)
Knee flexion (180 degrees)	19.6 ± 5.5	23.8 ± 6.6	−3.0 (−5.3, −0.7)	18.3 ± 5.5	22.0 ± 4.7	−2.4 (−4.6, −0.2)
Knee extension TBW (60 degrees)	151.8 ± 24.2	160.2 ± 28.4	−3.2 (−13.3, 6.9)	157.3 ± 32.9	166.4 ± 27.8	−1.8 (−15.2, 11.5)
Knee extension TBW (180 degrees)	125.6 ± 18.1	133.1 ± 20.5	−4.0 (−11.6, 3.5)	124.2 ± 24.3	133.0 ± 17.5	−3.4 (−12.8, 5.9)
Knee flexion TBW (60 degrees)	80.0 ± 18.8	91.0 ± 19.9	−8.2 (−16.0, −0.3)	76.3 ± 15.7	90.0 ± 12.8	−11.8 (−18.3, −5.4)
Knee flexion TBW (180 degrees)	72.2 ± 16.1	86.4 ± 12.3	−12.9 (−19.2, −6.6)	71.0 ± 19.5	81.9 ± 14.4	−9.3 (−17.1, −1.5)

Data are presented as absolute numbers (*n*) with proportions (%) and means ± SDs. We used ANCOVA to estimate mean differences (95% CIs) adjusted for age.

BMC = bone mineral content; BMD = bone mineral density; CI = confidence interval; DXA = dual‐energy X‐ray absorptiometry; n.a. = non‐applicable; SD = standard deviation.

### Development in musculoskeletal traits during the entire 15‐year study period

**Fig 2:**
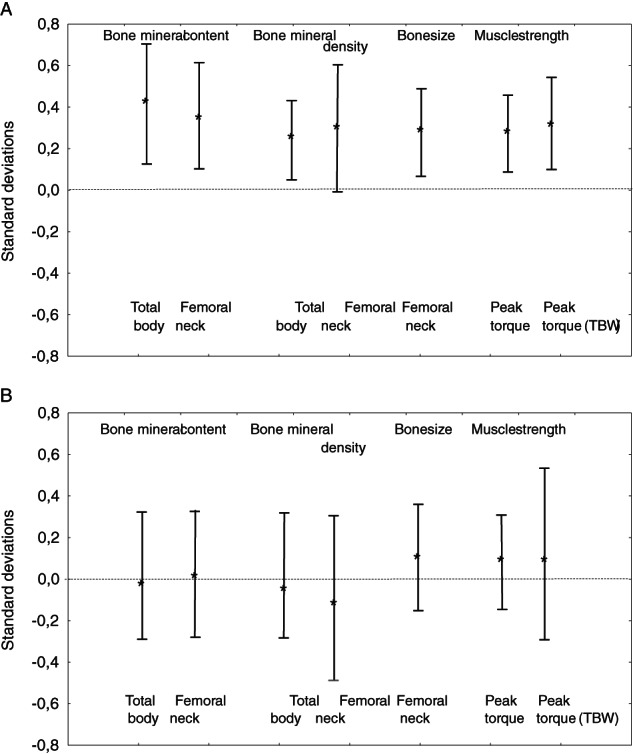
(*A*) Changes from baseline to 7 years after the intervention in children with daily school PA. (*B*) Changes from end of intervention to 7 years after the intervention in children with daily school PA. Gain in BMC and BMD in total body less head and femoral neck, gain in bone size in femoral neck, and gain in muscle strength in knee flexion peak torque 180 degrees per second in absolute values and relative to body weight (adjusted for sex and follow‐up period) in the intervention group expressed in SDs, compared to mean gain in the control group (0.0 SD) with the bars representing the 95% CIs. (*A*) Represents gain from baseline to follow‐up and (*B*) from end of the intervention to follow‐up. BMC = bone mineral content; BMD = bone mineral density; CI = confidence interval; SD = standard deviation.

During the entire 15‐year study period, we found greater gain in BMC (mean difference in total body less head +182.5 g [95% CI, 55.1 to 309.9]), BMD (mean difference in total body less head +0.03 g/cm^2^ [95% CI, 0.003 to 0.05]), bone size (femoral neck area + 0.2 cm^2^ [95% CI, 0.1 to 0.4]), and muscle strength (PT_flex60_ + 9.2 Nm [95% CI, 2.9 to 15.5]) in the intervention than in the control group (Table 2). This corresponds to +0.4 (95% CI, 0.1 to 0.7) SD higher gain in BMC, +0.3 (95% CI, 0.04 to 0.5) SD higher gain in BMD, +0.3 (95% CI, 0.1 to 0.5) SD higher gain in bone size, and +0.3 (95% CI, 0.1 to 0.5) SD higher gain in muscle strength (Fig. 2*A*). Interaction terms revealed that intervention was associated with greater effect in girls than in boys regarding BMD gain (*p* < 0.05 for gain in total body less head and leg), but similar development regarding bone size and muscle strength gain.

**Table 2:** Differences in Gain in Soft Tissue Composition and Musculoskeletal Traits Measured With DXA and Biodex, From Baseline to Follow‐Up, and From End of Intervention to Follow‐Up

Parameter	Gain from baseline (mean age 8 ± 17 years) to 7 years after the intervention terminated (mean age 23 ± 2 years) (*n* = 209)				Gain from end of the intervention (mean age 15 ± 1 years) to 7 years after the intervention terminated (mean age 23 ± 2 years) (*n* = 161)			
	Intervention (*n* = 131)	Control (*n* = 78)	Mean difference (adjusted analysis)	*p* (adjusted analysis)	Intervention (*n* = 108)	Control (*n* = 53)	Mean difference (adjusted analysis)	*p* (adjusted analysis)
Follow‐up period (years)	15.1 ± 2.0	14.2 ± 1.9	n.a.	n.a.	7.4 ± 2.1	6.7 ± 2.2	n.a.	n.a.
Anthropometry (kg)								
Total body fat mass	16.4 (15.0, 17.7)	14.9 (13.2, 16.6)	1.4 (−0.8, 3.6)	0.20	6.7 (5.6, 7.9)	7.6 (6.0, 9.2)	−0.9 (−2.8, 1.1)	0.39
Total body lean mass	29.0 (28.0, 30.1)	27.8 (26.5, 29.1)	1.2 (−0.4, 2.9)	0.15	4.8 (3.9, 5.8)	4.1 (2.8, 5.4)	0.8 (−0.9, 2.4)	0.35
BMC (g)								
Total body less head	**1749.1 (1672.5, 1825.7)**	**1586.8 (1491.3 1682.4)**	**182.5 (55.1, 309.9)**	**0.005**	175.9 (121.5, 230.4)	186.0 (108.2, 263.8)	−10.1 (−105.6, 85.5)	0.84
Arms	302.0 (292.6, 311.4)	297.7 (285.8, 309.6)	4.2 (−11.1, 19.7)	0.58	102.1 (93.0, 111.3)	102.6 (89.5, 115.7)	−0.5 (−16.5, 15.6)	0.96
Legs	**800.8 (776.4, 825.3)**	**759.5 (728.5, 790.4)**	**41.4 (1.4, 81.3)**	**0.04**	117.2 (96.6, 137.7)	127.1 (97.7, 156.4)	−9.9 (−45.9, 26.2)	0.59
Spine	**133.5 (127.6, 139.4)**	**117.5 (109.9, 125.0)**	**16.0 (6.3, 25.7)**	**0.001**	−53.2 (−62.3, −44.1)	−56.7 (−69.7, −43.7)	3.5 (−12.4, 19.5)	0.66
Hip – femoral neck	**3.0 (2.9, 3.2)**	**2.7 (2.5, 2.9)**	**0.3 (0.1, 0.6)**	**0.007**	0.2 (0.1, 0.4)	0.2 (0.0, 0.4)	0.0 (−0.2, 0.3)	0.94
Hip – Wards triangle	**1.8 (1.7, 1.9)**	**1.5 (1.4, 1.7)**	**0.3 (0.03, 0.5)**	**0.03**	−0.2 (−0.3, 0.0)	−0.3 (−0.5, −0.1)	0.1 (−0.2, 0.4)	0.45
BMD (g/cm^2^)								
Total body less head	**0.40 (0.38, 0.41)**	**0.37 (0.35, 0.39)**	**0.03 (0.003, 0.05)**	**0.02**	0.05 (0.03, 0.06)	0.05 (0.03, 0.07)	0.00 (−0.03, 0.02)	0.77
Arms	**0.19 (0.18, 0.20)**	**0.17 (0.15, 0.18)**	**0.02 (0.01, 0.04)**	**0.01**	−0.04 (−0.06, −0.03)	−0.05 (−0.07, −0.03)	0.01 (−0.04, 0.05)	0.48
Legs	**0.56 (0.55, 0.58)**	**0.52 (0.50, 0.55)**	**0.04 (0.01, 0.07)**	**0.006**	0.10 (0.09, 0.12)	0.10 (0.08, 0.12)	0.00 (−0.02, 0.03)	0.88
Spine	**0.43 (0.41, 0.45)**	**0.39 (0.37, 0.42)**	**0.03 (0.00, 0.06)**	**0.02**	0.08 (0.07, 0.10)	0.09 (0.07, 0.12)	−0.01 (−0.04, 0.02)	0.55
Hip – femoral neck	0.35 (0.33, 0.37)	0.32 (0.29, 0.34)	0.04 (−0.00, 0.07)	0.06	0.05 (0.03, 0.07)	0.06 (0.03, 0.08)	−0.01 (−0.04, 0.02)	0.60
Hip – Wards triangle	**0.21 (0.19, 0.24)**	**0.17 (0.13, 0.20)**	**0.05 (0.00, 0.09)**	**0.03**	−0.05 (−0.07, −0.03)	−0.06 (−0.08, −0.03)	0.00 (−0.03, 0.04)	0.81
Bone size (cm^2^)								
Hip – femoral neck	**1.6 (1.5, 1.7)**	**1.4 (1.3, 1.5)**	**0.2 (0.1, 0.4)**	**0.009**	0.0 (−0.1, 0.1)	−0.1 (−0.2, 0.1)	0.0 (−0.1, 0.2)	0.08
Peak torque muscle strength (Nm)								
Knee extension (60 degrees)	149.8 (142.7, 156.9)	141.3 (132.1, 150.6)	8.4 (−3.4, 20.3)	0.16	53.2 (45.9, 60.6)	55.4 (44.9, 65.8)	−2.1 (−15.0, 10.8)	0.75
Knee extension (180 degrees)	**102.4 (98.0, 106.7)**	**90.9 (85.2, 96.5)**	**11.5 (4.2, 18.8)**	**0.002**	34.1 (29.0, 39.3)	33.2 (25.9, 40.6)	0.9 (−8.1, 9.9)	0.85
Knee flexion (60 degrees)	**81.8 (78.0, 85.6)**	**72.6 (67.7, 77.5)**	**9.2 (2.9, 15.5)**	**0.004**	26.3 (21.9, 30.6)	24.0 (17.8, 30.2)	2.3 (−5.3, 9.9)	0.55
Knee flexion (180 degrees)	**55.1 (52.1, 58.0)**	**47.1 (43.4, 50.9)**	**7.9 (3.1, 12.8)**	**0.001**	13.9 (10.6, 17.2)	14.9 (10.3, 19.6)	−1.0 (−6.7, 4.8)	0.73
Peak torque muscle strength relative to total body weight (TBW)								
Knee extension TBW (60 degrees)	81.6 (74.1, 89.1)	84.0 (74.3, 93.7)	−2.4 (−14.9, 10.1)	0.70	11.9 (4.0, 19.8)	14.1 (2.9, 25.4)	−2.3 (−16.1, 11.6)	0.75
Knee extension TBW (180 degrees)	42.3 (37.9, 46.8)	36.1 (30.3, 41.8)	6.3 (−1.1, 13.7)	0.10	−5.3 (−15.6, 5.1)	0.8 (−13.9, 15.5)	−6.1 (−24.1, 12.0)	0.51
Knee flexion TBW (60 degrees)	**48.4 (44.1, 52.7)**	**38.1 (32.5, 43.6)**	**10.3 (3.2, 17.5)**	**0.005**	−0.9 (−6.4, 4.5)	−2.7 (−10.5, 5.1)	1.8 (−7.8, 11.4)	0.71
Knee flexion TBW (180 degrees)	**19.9 (16.1, 23.6)**	**8.8 (3.9, 13.6)**	**11.1 (4.9, 17.3)**	**<0.001**	−11.1 (−17.8, −5.8)	−5.4 (−13.9, 3.2)	−6.4 (−16.9, 4.0)	0.23

Of the individuals in this study, 48 did not participate in the last evaluation during the intervention period. Data are presented as absolute numbers (*n*) and means ± SDs. We used ANCOVA to estimate mean differences (95% CIs) adjusted for duration of follow‐up period and sex. Statistically significant group differences are in bold.

BMC = bone mineral content; BMD = bone mineral density; CI = confidence interval; DXA = dual‐energy X‐ray absorptiometry; n.a. = non‐applicable; SD = standard deviation.

### Changes in musculoskeletal traits during the 7‐year period following termination of the intervention

We found no attenuation of the musculoskeletal benefits in children with daily school PA during the first 7 years after termination of intervention (all group comparisons *p* > 0.05) (Table 2, Fig. 2*B*). Interaction terms pointed to similar musculoskeletal development for boys and girls during this period (all *p* > 0.05).

### Sex‐specific group characteristics 7 years after termination of the intervention

Sex‐specific group characteristics 7 years after termination of the intervention program are presented in Supplemental Appendix 2.

## Discussion

In the POP cohort we have previously reported that a 9‐year daily school PA intervention program is associated with higher gain in bone mass and muscle strength compared to children with the standard amount of school PA of one to two lessons per school week^(^ ^7^, ^8^ ^)^; each year with more PA was accompanied by lower relative fracture incidence.^(^ ^7^ ^)^ In the current study, 7 years after termination of the intervention, we found that the musculoskeletal benefits remained without evidence of post‐termination attenuation. Because the benefits remain in adulthood, the outcome of the intervention seems even more beneficial. The study thus infers that it is possible to achieve long‐term benefits in bone mass and muscle strength by PA interventions at the population level during growth.

Some earlier reports infer that bone mass benefits from PA during young years may remain even after a reduction in PA.^(^ ^16^, ^17^, ^18^, ^19^, ^20^ ^)^ Findings of lower adult fracture incidence in individuals with high level of PA in childhood support this view.^(^ ^16^, ^17^, ^18^, ^19^ ^)^ Other reports oppose this view, finding that cessation of high PA is followed by greater bone mass loss than expected with aging.^(^ ^19^, ^24^, ^25^ ^)^ One drawback of the cited studies is that they include individuals with a self‐chosen high level of PA^(^ ^16^, ^17^, ^18^, ^19^, ^20^, ^24^, ^25^ ^)^ and with no bone mass values presented before the activity was initiated. It is not unlikely that these individuals had genetically determined beneficial bone mass, muscle strength, and physical ability, associated with success in sports. In contrast, the risk of selection bias in our study seems minimal, because children in the intervention group at study start were similar to children in the control group regarding bone mass and muscle strength.

Not only bone mass but also bone size correlates with the structural strength of the bone,^(^ ^27^, ^28^, ^33^ ^)^ and both bone mass and bone size are independent predictors of fractures.^(^ ^34^ ^)^ Benefits in bone size are then of great importance as the resistance to bending of a tubular structure; ie, bone, is proportional to the fourth power of the distance from the neutral axis.^(^ ^35^ ^)^ Some studies also infer that exercise‐induced benefits in bone mass are lost through time, whereas bone structure/bone size benefits remain,^(^ ^28^, ^29^, ^30^ ^)^ but we found residual benefits in both bone mass and bone size even 7 years after the program terminated.

The beneficial gain seems be driven by effects in girls (Supplemental Appendix 2). A possible explanation is that girls in general, as was also observed in our cohort, participate less in leisure‐time physical activity than boys.^(^ ^6^, ^7^, ^8^, ^36^ ^)^ The daily school PA program therefore contributed to a relatively greater increase in the total amount of PA in girls.^(^ ^6^, ^36^ ^)^ This sex difference may become even more prominent in puberty, because girls usually reduce PA more than boys in this period,^(^ ^37^ ^)^ as also in our cohort.^(^ ^6^, ^7^, ^8^, ^36^ ^)^

We are unable to draw causal inferences regarding high PA in childhood and high bone mass and superior muscle strength in adulthood, because the participants in the former intervention group voluntarily continued with more PA after the program.^(^ ^36^ ^)^ The lack of attenuation in muscle strength after termination of the program does not oppose this view, because muscle strength is known to adapt quickly to current level of PA.^(^ ^38^ ^)^ We are also unable to exclude factors other than the increased PA to influence the outcome. Because of the focus on PA, the children (and their parents) in the intervention school may have developed a greater knowledge of health‐related issues and by this consciously or unconsciously changed their habits. Children in the intervention group may thus improve their nutritional intake, cycle to school instead of taking the bus, and/or take the stairs instead of the elevator. However, we are not aware of any prospective controlled study that infer this causal relationship. Furthermore, from a clinical perspective, this is a minor concern, because the intervention reached the ultimate goal.

Study strengths include the prospective, controlled, population‐based study design, a period examined from childhood to adulthood, and data was available before the intervention was initiated. Study limitations include the small sample size and the high dropout frequency, with risks for selection bias and type II error. However, the dropout analyses oppose selection bias, and we compared all children together and not by sex to reduce the risk of type II error. A longer postintervention follow‐up period would have been beneficial, because we do not know whether the benefits remain in a longer perspective. Because most children were of Caucasian ethnicity, living in a middle‐class area, it is questionable if inferences can be transferred to other ethnic groups and/or socioeconomic settings. The lack of individual randomization is another weakness, but the schools refused this at study start due to practical problems with schedules. Further limitations include lack of objectively measured amount of PA.

We conclude that benefits in musculoskeletal gain achieved by daily school PA^(^ ^6^, ^7^, ^8^ ^)^ remain in young adulthood, 7 years after termination of the intervention program, without attenuation. Daily school PA seems to be one strategy on population‐based level to counteract low bone mass and inferior muscle strength in young adulthood.

## Conflict of Interest

None of the authors have any conflict of interest.

### PEER REVIEW

The peer review history for this article is available at https://publons.com/publon/10.1002/jbm4.10566.

## Supporting information

Additional supporting information may be found online in the Supporting Information section.

**Appendix 1**. The second dropout analyses comparing baseline values (at school start) between participants who attended the baseline and the seven‐year post‐intervention exam with the participants who attended only the baseline but not the seven‐year post‐intervention exam. Anthropometry was estimated with standard equipment, bone mineral content (BMC), bone mineral density (BMD), bone size and soft tissue composition with dual‐energy X‐ray absorptiometry (DXA) and muscle strength with Biodex®. Data presented as absolute numbers (n) and means ± standard deviations.

**Appendix 2**. Anthropometry, lifestyle characteristics, soft tissue composition and musculoskeletal traits measured with dual‐energy X‐ray absorptiometry (DXA) and with Biodex® 7 ± 2 years after the intervention terminated. Data presented as absolute numbers (n) with proportions (%) and means ± standard deviations. Mean differences (95% confidence intervals) and p‐values are adjusted for differences in age in ANCOVA analyses. Statistically significant group differences are bolded. Not applicable (n.a.).
