# Supplementary material for: Physical Activity at Growth Induces Bone Mass Benefits Into Adulthood – A Fifteen‐Year Prospective Controlled Study
Source: JBMR Plus. 2021 Nov 26;6(1):e10566. doi: 10.1002/jbm4.10566 (PMC8770997; doi:10.1002/jbm4.10566)
Supplement: Supplementary file 1 — Appendix 1. The second dropout analyses comparing baseline values (at school start) between participants who attended the baseline and the seven‐year post‐intervention exam with the participants who attended only the baseline but not the seven‐year post‐intervention exam. Anthropometry was estimated with standard equipment, bone mineral content (BMC), bone mineral density (BMD), bone size and soft tissue composition with dual‐energy X‐ray absorptiometry (DXA) and muscle strength with Biodex®. Data presented as absolute numbers (n) and means ± standard deviations. [file JBM4-6-e10566-s002.docx]

**Appendix 1.** The second dropout analyses comparing baseline values (at school start) between participants who attended the baseline and the seven-year post-intervention exam with the participants who attended only the baseline but not the seven-year post-intervention exam. Anthropometry was estimated with standard equipment, bone mineral content (BMC), bone mineral density (BMD), bone size and soft tissue composition with dual-energy X-ray absorptiometry (DXA) and muscle strength with Biodex®. Data presented as absolute numbers (n) and means ± standard deviations.

|  | **Boys (n=191)** | | |  | **Girls (n=158)** | | |
| --- | --- | --- | --- | --- | --- | --- | --- |
|  | **Participants**  **(n=108)** | **Dropouts**  **(n=83)** | **p-values** |  | **Participants**  **(n=101)** | **Dropouts**  **(n=57)** | **p-values** |
| **At school start (baseline)** |  |  |  |  |  |  |  |
| **Age** | 7.7 ± 0.6 | 7.8 ± 0.6 | 0.53 |  | 7.7 ± 0.7 | 7.5 ± 0.5 | 0.03 |
| **Anthropometry** |  |  |  |  |  |  |  |
| Height (cm) | 128.8 ± 6.6 | 129.0 ± 6.4 | 0.83 |  | 128.0 ± 6.8 | 128.0 ± 7.3 | 0.97 |
| Weight (kg) | 27.6 ± 5.4 | 28.0 ± 5.9 | 0.63 |  | 26.9 ± 5.2 | 28.0 ± 5.6 | 0.22 |
| BMI (kg/m^2^) | 16.5 ± 2.3 | 16.7 ± 2.5 | 0.63 |  | 16.3 ± 2.2 | 17.0 ± 2.8 | 0.08 |
| **Soft tissue composition (kg)** |  |  |  |  |  |  |  |
| Total body fat mass | 3.7 ± 3.0 | 4.2 ± 3.6 | 0.33 |  | 4.8 ± 2.9 | 5.6 ± 4.0 | 0.17 |
| Total body lean mass | 21.5 ± 3.0 | 21.6 ± 2.9 | 0.95 |  | 19.8 ± 2.5 | 20.2 ± 2.3 | 0.39 |
| **Bone mineral content (BMC; g)** |  |  |  |  |  |  |  |
| Total body less head | 651.2 ± 148.2 | 658.8 ± 161.0 | 0.74 |  | 613.5 ± 139.5 | 628.9 ± 135.4 | 0.51 |
| Arms | 88.2 ± 19.6 | 87.1 ± 20.5 | 0.69 |  | 80.2 ± 18.3 | 80.9 ± 16.0 | 0.80 |
| Legs | 280.0 ± 70.8 | 288.0 ± 74.1 | 0.45 |  | 272.6 ± 65.7 | 281.6 ± 67.0 | 0.43 |
| Spine | 84.9 ± 19.4 | 85.1 ± 22.2 | 0.93 |  | 79.5 ± 17.6 | 82.5 ± 18.5 | 0.33 |
| Hip – femoral neck | 2.8 ± 0.7 | 2.9 ± 0.6 | 0.69 |  | 2.6 ± 0.7 | 2.6 ± 0.5 | 0.61 |
| Hip – Wards triangle | 1.3 ± 0.6 | 1.3 ± 0.4 | 0.72 |  | 1.2 ± 0.5 | 1.1 ± 0.3 | 0.66 |
| **Bone mineral density (BMD; g/cm^2^)** | | | | | | | |
| Total body less head | 0.69 ± 0.05 | 0.69 ± 0.06 | 0.97 |  | 0.68 ± 0.05 | 0.69 ± 0.05 | 0.48 |
| Arms | 0.62 ± 0.05 | 0.61 ± 0.04 | 0.35 |  | 0.60 ± 0.05 | 0.60 ± 0.04 | 0.78 |
| Legs | 0.75 ± 0.07 | 0.76 ± 0.07 | 0.94 |  | 0.75 ± 0.07 | 0.76 ± 0.07 | 0.45 |
| Spine | 0.68 ± 0.06 | 0.68 ± 0.07 | 0.99 |  | 0.69 ± 0.06 | 0.69 ± 0.06 | 0.84 |
| Hip – femoral neck | 0.78 ± 0.11 | 0.78 ± 0.11 | 0.81 |  | 0.71 ± 0.10 | 0.73 ± 0.08 | 0.30 |
| Hip – Wards triangle | 0.81 ± 0.13 | 0.83 ± 0.13 | 0.46 |  | 0.74 ± 0.14 | 0.75 ± 0.10 | 0.86 |
| **Bone size (cm^2^)** |  |  |  |  |  |  |  |
| Hip – femoral neck | 3.6 ± 0.5 | 3.7 ± 0.4 | 0.67 |  | 3.6 ± 0.5 | 3.6 ± 0.4 | 0.90 |
| **Peak torque muscle strength (Nm)** | | | | | | | |
| Knee extension (60°) | 42.0 ± 10.2 | 44.0 ± 11.6 | 0.21 |  | 42.6 ± 11.1 | 42.2 ± 9.4 | 0.80 |
| Knee extension (180°) | 34.7 ± 8.0 | 35.9 ± 9.1 | 0.34 |  | 33.8 ± 8.2 | 33.9 ± 6.1 | 0.94 |
| Knee flexion (60°) | 22.9 ± 7.0 | 23.5 ± 7.1 | 0.52 |  | 21.9 ± 5.9 | 21.8 ± 5.2 | 0.95 |
| Knee flexion (180°) | 20.9 ± 6.2 | 21.3 ± 6.0 | 0.69 |  | 19.9 ± 5.5 | 19.1 ± 5.0 | 0.37 |
| **Peak torque muscle strength relative to total body weight (TBW)** | | | | | | | |
| Knee extension TBW (60°) | 154.5 ± 25.8 | 160.0 ± 29.3 | 0.20 |  | 160.9 ± 30.9 | 154.1 ± 29.6 | 0.19 |
| Knee extension TBW (180°) | 128.0 ± 19.1 | 130.8 ± 25.3 | 0.39 |  | 127.8 ± 21.9 | 124.0 ± 18.8 | 0.28 |
| Knee flexion TBW (60°) | 83.6 ± 19.7 | 85.9 ± 22.5 | 0.46 |  | 82.3 ± 16.1 | 79.8 ± 17.2 | 0.37 |
| Knee flexion TBW (180°) | 76.8 ± 16.3 | 78.7 ± 22.1 | 0.49 |  | 75.8 ± 18.2 | 70.0 ± 17.5 | 0.06 |
