# Supplementary material for: Physical Activity at Growth Induces Bone Mass Benefits Into Adulthood – A Fifteen‐Year Prospective Controlled Study
Source: JBMR Plus. 2021 Nov 26;6(1):e10566. doi: 10.1002/jbm4.10566 (PMC8770997; doi:10.1002/jbm4.10566)
Supplement: Supplementary file 2 — Appendix 2. Anthropometry, lifestyle characteristics, soft tissue composition and musculoskeletal traits measured with dual‐energy X‐ray absorptiometry (DXA) and with Biodex® 7 ± 2 years after the intervention terminated. Data presented as absolute numbers (n) with proportions (%) and means ± standard deviations. Mean differences (95% confidence intervals) and p‐values are adjusted for differences in age in ANCOVA analyses. Statistically significant group differences are bolded. Not applicable (n.a.). [file JBM4-6-e10566-s001.docx]

**Appendix 2** Anthropometry, lifestyle characteristics, soft tissue composition and musculoskeletal traits measured with dual-energy X-ray absorptiometry (DXA) and with Biodex® 7 ± 2 years after the intervention terminated. Data presented as absolute numbers (n) with proportions (%) and means ± standard deviations. Mean differences (95% confidence intervals) and p-values are adjusted for differences in age in ANCOVA analyses. Statistically significant group differences are bolded. Not applicable (n.a.)

|  | **Men (n=108)** | | | |  | **Women (n=101)** | | | | |
| --- | --- | --- | --- | --- | --- | --- | --- | --- | --- | --- |
|  | **Intervention**  **(n=72)** | **Control**  **(n=36)** | **Mean difference**  **(adjusted)** | **P value**  **(adjusted)** |  | **Intervention**  **(n=59)** | **Control**  **(n=42)** | | **Mean difference**  **(adjusted)** | **P value**  **(adjusted)** |
| **Age (years)** | 22.5 ± 2.2 | 22.1 ± 2.2 | n.a. | 0.29 |  | 22.9 ± 1.9 | 22.4 ± 2.0 | | n.a. | 0.15 |
| **Anthropometry** |  |  |  |  |  |  |  | |  |  |
| Height (cm) | 180.6 ± 7.0 | 180.9 ± 8.2 | -0.4 (-3.4, 2.6) | 0.81 |  | 168.8 ± 5.5 | 167.5 ± 6.4 | | 1.0 (-1.4, 3.4) | 0.40 |
| Weight (kg) | 79.6 ± 14.6 | 77.8 ± 12.1 | 1.6 (-4.0, 7.3) | 0.57 |  | 66.7 ± 11.5 | 63.6 ± 12.5 | | 3.0 (-1.9, 7.8) | 0.23 |
| BMI (kg/m^2^) | 24.4 ± 4.1 | 23.8 ± 3.2 | 0.5 (-1.0, 2.1) | 0.46 |  | 23.4 ± 3.9 | 22.6 ± 3.7 | | 0.8 (-0.7, 2.4) | 0.30 |
| **Lifestyle [n (%)]** |  |  |  |  |  |  |  | |  |  |
| Exclusion of dairy products | 4/72 (6%) | 1/36 (3%) | n.a. | 0.52 |  | 4/59 (7%) | 4/42 (10%) | | n.a. | 0.61 |
| Any medical condition | 19/72 (25%) | 4/36 (11%) | n.a. | 0.07 |  | 13/59 (22%) | 6/42 (14%) | | n.a. | 0.19 |
| Current medication | 2/72 (3%) | 1/36 (3%) | n.a. | 1.00 |  | 5/59 (8%) | 1/42 (2%) | | n.a. | 0.20 |
| Birth control pills | n.a. | n.a. | n.a. | n.a. |  | **23/59 (39%)** | **26/42 (62%)** | | n.a. | **0.02** |
| Teetotaler | 3/72 (4%) | 1/35 (3%) | n.a. | 0.74 |  | 2/59 (3%) | 3/42 (7%) | | n.a. | 0.39 |
| Smoker | 3/72 (4%) | 4/35 (11%) | n.a. | 0.15 |  | 11/59 (19%) | 11/42 (26%) | | n.a. | 0.37 |
| **Physical activity** (**hours/week**) | | | | | | | | | | |
| Total organized PA (hours/week) | 5.8 ± 5.6 | 5.0 ± 3.3 | 0.9 (-1.2, 3.0) | 0.38 |  | 5.1 ± 4.9 | 4.4 ± 2.9 | | 0.6 (-1.2, 2.4) | 0.49 |
| **Soft tissue composition (kg)** | | | | | | | | | | |
| Total body fat mass | 18.8 ± 9.5 | 17.2 ± 6.4 | 1.4 (-2.2, 4.9) | 0.44 |  | 22.8 ± 8.0 | 21.1 ± 8.7 | | 1.7 (-1.8, 5.2) | 0.33 |
| Total body lean mass | 58.1 ± 8.0 | 57.3 ± 8.3 | 0.7 (-2.7, 4.1) | 0.68 |  | 41.2 ± 5.0 | 40.2 ± 5.3 | | 0.8 (-1.3, 2.9) | 0.47 |
| **Bone mineral content (BMC; g)** | | | | | | | | | | |
| Total body less head | 2634.7 ± 456.9 | 2608.8 ± 382.7 | 24.3 (-155.8, 204.5) | 0.79 |  | **2024.1 ± 264.7** | | **1897.8 ± 259.6)** | **123.8 (13.9, 233.7)** | **0.03** |
| Arms | 459.5 ± 79.7 | 453.7 ± 67.5 | 3.0 (-28.0, 33.9) | 0.85 |  | 315.9 ± 41.3 | 307.7 ± 39.9 | | 6.5 (-10.4, 23.4) | 0.45 |
| Legs | 1216.8 ± 201.3 | 1210.1 ± 175.4 | 5.8 (-74.4, 86.0) | 0.89 |  | 932.2 ± 130.3 | 882.4 ± 123.5 | | 47.8 (-5.5, 101.1) | 0.08 |
| Spine | 230.8 ± 53.3 | 225.5 ± 41.1 | 7.2 (-13.0, 27.4) | 0.48 |  | **198.2 ± 31.8** | **180.5 ± 33.4** | | **18.6 (5.1, 32.1)** | **0.008** |
| Hip – total hip | 41.5 ± 7.6 | 41.8 ± 6.3 | -0.3 (-3.3, 2.6) | 0.84 |  | 33.6 ± 5.1 | 31.8 ± 5.2 | | 1.7 (-0.4, 3.9) | 0.11 |
| Hip – femoral neck | 6.2 ± 1.2 | 6.2 ± 1.0 | 0.0 (-0.4, 0.5) | 0.93 |  | 5.1 ± 0.7 | 4.8 ± 0.8 | | 0.3 (-0.0, 0.6) | 0.09 |
| Hip – Wards triangle | 3.5 ± 1.0 | 3.4 ± 0.8 | 0.1 (-0.1, 0.4) | 0.24 |  | 2.4 ± 0.5 | 2.3 ± 0.6 | | 0.1 (-0.1, 0.4) | 0.24 |
| **Bone mineral density (BMD; g/cm^2^)** | | | | | | | | | | |
| Total body less head | 1.13 ± 0.13 | 1.14 ± 0.10 | 0.00 (-0.05, 0.05) | 0.88 |  | **1.03 ± 0.08** | **0.99 ± 0.08** | | **0.05 (0.01, 0.08)** | **0.007** |
| Arms | 0.85 ± 0.11 | 0.84 ± 0.09 | 0.02 (-0.02, 0.06) | 0.36 |  | **0.74 ± 0.06** | **0.71 ± 0.06** | | **0.03 (0.0, 0.05)** | **0.03** |
| Legs | 1.39 ± 0.15 | 1.38 ± 0.13 | 0.00 (-0.06, 0.06) | 0.98 |  | **1.24 ± 0.11** | **1.19 ± 0.10** | | **0.05 (0.01, 0.10)** | **0.02** |
| Spine | 1.14 ± 0.16 | 1.14 ± 0.12 | 0.00 (-0.06, 0.06) | 0.97 |  | **1.09 ± 0.12** | **1.03 ± 0.11** | | **0.06 (0.01, 0.11)** | **0.03** |
| Hip – femoral neck | 1.11 ± 0.17 | 1.13 ± 0.16 | -0.02 (-0.09, 0.05) | 0.58 |  | **1.07 ± 0.12** | **1.02 ± 0.14** | | **0.06 (0.00, 0.11)** | **0.04** |
| Hip – Wards triangle | 1.01 ± 0.20 | 1.03 ± 0.18 | -0.01 (-0.09, 0.07) | 0.73 |  | 0.95 ± 0.13 | 0.90 ± 0.15 | | 0.05 (-0.00, 0.11) | 0.07 |
| **Bone size (cm^2^)** |  |  |  |  |  |  |  | |  |  |
| Hip – femoral neck | 5.6 ± 0.4 | 5.5 ± 0.4 | 0.1 (-0.1, 0.3) | 0.26 |  | 4.8 ± 0.3 | 4.7 ± 0.3 | | 0.0 (-0.1, 0.1) | 0.97 |
| **Peak torque muscle strength (Nm)** | | | | | | | | | | |
| Knee extension (60°) | 225.9 ± 49.0 | 234.8 ± 54.9 | -5.8 (-26.1, 14.5 | 0.57 |  | 150.2 ± 37.5 | 148.2 ± 39.7 | | 8.6 (-3.8, 21.1) | 0.17 |
| Knee extension (180°) | 161.2 ± 34.3 | 159.6 ± 35.4 | 5.0 (-8.5, 18.1) | 0.45 |  | 104.2 ± 22.4 | 103.3 ± 28.1 | | 5.6 (2.1, 13.4) | 0.15 |
| Knee flexion (60°) | 123.2 ± 27.8 | 127.4 ± 30.9 | -1.6 (-12.7, 9.4) | 0.77 |  | **77.8 ± 20.1** | **75.0 ± 24.1** | | **7.3 (1.1, 13.6)** | **0.02** |
| Knee flexion (180°) | 88.7 ± 22.7 | 91.4 ± 21.2 | -0.8 (-9.4, 7.8) | 0.85 |  | **56.2 ± 13.2** | **53.9 ± 17.7** | | **5.3 (0.7, 9.9)** | **0.02** |
| **Peak torque muscle strength relative to total body weight (TBW)** | | | | | | | | | | |
| Knee extension TBW (60°) | 269.0 ± 45.4 | 276.8 ± 45.8 | -10.4 (-28.6, 7.8) | 0.26 |  | 209.3 ± 35.2 | 212.8 ± 39.5 | | -4.8 (-19.8, 10.1) | 0.52 |
| Knee extension TBW (180°) | 191.6 ± 29.0 | 187.8 ± 26.4 | 3.3 (-8.3, 14.8) | 0.58 |  | 145.6 ± 20.0 | 147.2 ± 22.6 | | -2.5 (-11.0, 6.0) | 0.56 |
| Knee flexion TBW (60°) | 146.5 ± 25.3 | 149.3 ± 21.9 | -3.2 (-13.1, 6.8) | 0.53 |  | 108.8 ± 21.1 | 105.9 ± 18.6 | | 3.4 (-4.7, 11.6) | 0.40 |
| Knee flexion TBW (180°) | 105.5 ± 21.4 | 107.6 ± 16.5 | -2.4 (-10.6, 5.9) | 0.57 |  | 78.8 ± 14.7 | 76.3 ± 14.1 | | 2.6 (-3.3, 8.4) | 0.39 |
